# Effects of urease and nitrification inhibitors on soil N, nitrifier abundance and activity in a sandy loam soil

**DOI:** 10.1007/s00374-019-01411-5

**Published:** 2019-11-25

**Authors:** Qingling Fu, Maïder Abadie, Aimeric Blaud, Alison Carswell, Tom H. Misselbrook, Ian M. Clark, Penny R. Hirsch

**Affiliations:** 1 Sustainable Agriculture Sciences Department, Rothamsted Research, Harpenden, AL5 2JQ Hertfordshire UK; 2 College of Resources and Environment, Huazhong Agricultural University, Wuhan, 430070 Hubei Province People’s Republic of China; 3 Present Address: School of Applied Sciences, Edinburgh Napier University, Sighthill Campus, Edinburgh, EH11 4BN UK; 4 Sustainable Agriculture Sciences Department, Rothamsted Research, North Wyke, Okehampton, EX20 2SB Devon UK

**Keywords:** Urea fertilizer, Urease inhibitor, Nitrification inhibitor, Arable soil, Soil microbial diversity, Nitrification genes

## Abstract

Inhibitors of urease and ammonia monooxygenase can limit the rate of conversion of urea to ammonia and ammonia to nitrate, respectively, potentially improving N fertilizer use efficiency and reducing gaseous losses. Winter wheat grown on a sandy soil in the UK was treated with urea fertilizer with the urease inhibitor N-(n-butyl) thiophosphoric triamide (NBPT), the nitrification inhibitor dicyandiamide (DCD) or a combination of both. The effects on soil microbial community diversity, the abundance of genes involved in nitrification and crop yields and net N recovery were compared. The only significant effect on N-cycle genes was a transient reduction in bacterial ammonia monooxygenase abundance following DCD application. However, overall crop yields and net N recovery were significantly lower in the urea treatments compared with an equivalent application of ammonium nitrate fertilizer, and significantly less for urea with DCD than the other urea treatments.

**Electronic supplementary material:**

The online version of this article (10.1007/s00374-019-01411-5) contains supplementary material, which is available to authorized users.

## Introduction

Nitrogen fertilizer is required for arable crop production but nitrous oxide (N_2_O) losses due to both microbial activity and abiotic processes are a major environmental concern and a challenge for sustainable agriculture. Fertilizers that do not contain nitrate (the substrate for denitrification) are rapidly converted to nitrate in soil. Urea, globally the most commonly used N fertilizer, is subject to hydrolysis by the action of microbial urease to generate ammonia which can be lost by volatilization or oxidized to nitrate by microbial nitrifiers. Various chemical compounds have been assessed for their effectiveness in reducing ammonia emissions from urea fertilizer through their inhibition of the urea hydrolysis process (e.g. Silva et al. 2017), in reducing N_2_O emissions from urea and ammonia-based fertilizers through their inhibition of the nitrification process (e.g. Akiyama et al. 2010; Gilsanz et al. 2016), and the consequent impacts on crop yield and N use efficiency (e.g. Abalos et al. 2014; Rose et al. 2018). The urease inhibitor (UI), N-(n-butyl) thiophosphoric triamide (NBPT) occupies the active sites in urease and is the basis of commercial products that are applied together with urea fertilizers (Sigurdarson et al. 2018). NBPT is reported to delay the hydrolysis of urea fertilizer by 7 to 10 days (Zaman et al. 2008), resulting in a smaller pH increase around the urea granule than for urea alone, and hence lower ammonia volatilization losses. Dicyandiamide (DCD) is a nitrification inhibitor (NI) that slows oxidation of ammonia-N to nitrate-N by deactivating the bacterial ammonia monooxygenease, AMO (Amberger 2008). AMO-containing ammonia-oxidizing bacteria (AOB) and archaea (AOA) convert ammonia to hydroxylamine, which is further oxidized to nitrite (Prosser and Nicol 2012). Although denitrifying bacteria are thought to be the main source of N_2_O in arable soil, losses are also directly attributed to both AOA and AOB, which generate N_2_O by “nitrifier denitrification” (Wrage-Monnig et al. 2018). Also, hydroxylamine can decompose spontaneously to generate N_2_O and this process occurs in acid soils < pH 5.0 (Heil et al. 2016). The final step in nitrification is the conversion of nitrite to nitrate by nitrite-oxidizing bacteria (NOB) containing nitrite oxidoreductase (NXR), which includes the genera *Nitrobacter* and *Nitrospira.* The *Nitrospira* include a recently discovered group of “comammox” bacteria that contain AMO and can undertake complete nitrification, converting ammonia to nitrate (Daims et al. 2016). Two clades have been identified but only the AMO gene of comammox clade B was detected in soil (Pjevac et al. 2017).

The objective of this study was to evaluate the effects of NBPT and DCD used singly or in combination on soil mineral N dynamics and the functional genes involved in urea hydrolysis (*ureC*) and nitrification (*amoA*, *nxrA*). Genes for urease are relatively common in soil, produced by 17–30% of soil microorganisms (Lloyd and Sheaffe 1973). Reportedly, up to 50% of soil urease is extracellular (Klose and Tabatabai 1999; Qin et al. 2010), thus readily accessible to inhibitors. In contrast, AMO is membrane bound in both bacteria and archaea (Prosser and Nicol 2012) and the AOA and AOB together were found to comprise fewer than 1% of prokaryotes in an arable soil (Hirsch et al. 2017). The abundance of NOB and comammox bacteria in soil is uncertain but ammonia oxidation is usually considered to be the rate-limiting step in nitrification (Kowalchuk and Stephen 2001). In this study, we test the hypothesis that a combination of a UI and NI together with urea fertilizer applied to an arable crop is more efficient at delaying nitrification than either inhibitor alone. We measured soil N during the experiment and crop yields at the end of the season, and we monitored the responses of different soil microbial groups to the changes in soil mineral N, using qPCR with 16S rRNA gene diagnostic primers for Bacteria and Archaea, ITS sequence primers for Fungi as well for functional nitrification genes. Measuring gene abundance and activity in conjuntion with N-cycling in situ in the field should advance understanding of enzyme-mediated soil processes (Nannipieri et al. 2018).

Although there have been many studies on the combined effects of urease and nitrification inhibitors in the field, very few have attempted to relate this in the abundance and activity of the relevant microbial genes. This is the first report of how a combination of the commercially important inibitors DCD and NBPT together influence gene abundance and expression in the soil nitrifier community. This includes bacterial and archaeal ammonia oxidizers and nitrite-oxidizing bacteria in an arable soil after application of urea or ammonium nitrate fertilizer with different combinations of DCD and NBPT.

## Material and methods

### Experimental site

The experiment was conducted in 2017 with winter wheat at Horsepool field, Woburn in Bedfordshire UK, on a sandy loam soil classified as Cambric Arenosol (FAO 1990), pH 6.2, total N 1.84 g kg^−1^, total C 18.9 g kg^−1^, average annual rainfall 640 mm and soil temperature 10.4 °C (Johnston et al. 2017). Rainfall and soil temperature (surface and 10 cm depth) are monitored daily at Woburn. The field had previously been in an arable rotation, subsoiled after harvest in September 2013, with crops of spring barley in 2014 and 2015. Winter wheat var. Siskin was drilled in 2016.

### Experimental design

The field experiment consisted of six treatments: nil (zero N control); ammonium nitrate fertilizer (AN); urea fertilizer (urea); urea with 6500 mg kg^−1^ DCD incorporated (urea + NI); urea with 660 mg kg^−1^ NBPT coating (urea + UI); urea with DCD incorporated and NBPT coating (urea + NI + UI). There were six replicates of each treatment in a completely randomized design. The total fertilizer application rate to all plots apart from the zero N control was 200 kg N ha^−1^, considered the optimum rate for this site and wheat variety. This was applied as a split dose, 50 kg N ha^−1^, on March 6th, 100 kg N ha^−1^ on April 4th and 50 kg N ha^−1^ on May 3rd. The management of different treatments was identical apart from the different fertilizer/inhibitor combinations. Soil monitoring commenced on April 6th, 2 days after the highest dose was applied and > 4 weeks after the initial lower dose. For practical reasons, to keep numbers manageable, only four of the six replicate plots were sampled for mineral and microbiological analysis, and the AN treatment was not included although total N offtake and recovery in wheat grain and straw was calculated for all replicates and treatments.

Soil cores (5 cm diameter and 0–20 cm depth) were collected on April 6th, 12th and 19th (2, 8 and 15 days after urea application). Cores were processed straight away in the field and flash-frozen in liquid N within 3 min of collection. Processing of field samples included removal of stones, plant roots, fauna and debris, followed by sieving < 2 mm then placing in liquid N. Samples were stored at − 80 °C for subsequent molecular analysis. Subsamples were extracted in 2 M KCl (5 mL g^−1^ dw soil) by vigorous shaking (120 rpm) for 2 h then left to stand for 45 min before filtering through Whatman no 1 paper. Nitrate (NO_3_^−^) and ammonium (NH_4_^+^) in the filtrate were analysed simultaneously using a Skalar SAN^PLUS^ System continuous flow analyser; nitrite (NO_2_^−^) was measured in a separate Skalar run using less dilute soil extracts.

### Grain and straw yields

Plots were harvested on August 16th using a small plot harvester. Harvest weights of grain and straw per plot were recorded and subsamples of each taken for analyses of dry matter (DM) content by drying at 100 °C to constant weight and total N content using a Dumas combustion analyser (LECO).

### Nucleic acid extraction and 16S rRNA amplicon sequencing

DNA and RNA were co-extracted from the same 2-g frozen soil sample using the RNA PowerSoil® isolation kit and RNA PowerSoil® DNA Elution Accessory Kit (MO BIO Laboratories, Inc.) following a modification to the manufacturer’s instructions, whereby the 15-min shaking on a flatbed vortex was replaced by an alternative strategy, a 2 × 30-s bead beading step (5.5 m s^−1^, Fastprep). RNA samples were DNase treated to remove DNA contamination using the DNase Max Kit (Qiagen, Manchester, UK), following the manufacturer’s protocol. Direct PCRs were carried out on DNase-treated RNA to confirm all contaminating DNA had been removed. The quantity and quality of extracted DNA and DNase-treated RNA were analysed by fluorometer Qubit® 2.0 dsDNA and RNA BR Assay Kits and Nanodrop microvolume spectrophotometer (Thermo Fisher Scientific). Previously, we found bead-beating methods to reveal the greatest diversity in soil metagenomic DNA (Delmont et al. 2011).

Soil bacterial diversity was assessed at the first sampling point by next-generation sequencing of the V4–V5 region of 16S rRNA genes, assigning to operational taxonomic units (OTU) and performing non-metric multidimensional scaling (NMDS) as described previously (Hirsch et al. 2017) with the following modifications. The 16SrRNA gene high-throughput amplicon sequencing was performed at Novogene (HK) Co. Ltd. (Hong Kong, China) using an Illumina HiSeq platform with a paired-end read length of 250 bp and primers 515F (GTGYCAGCMGCCGCGGTAA, Parada et al. 2016) and 926R (CCGYCAATTYMTTTRAGTTT, Quince et al. 2011; Parada et al. 2016) as used by the Earth Microbiome project (http://press.igsb.anl.gov/earthmicrobiome/protocols-and-standards/16s/). Sequence data were analysed using QIIME 2 version 2018.11.0. Raw reads were quality checked, trimmed (removing primers, adapters, and the last 10 bp), merged using VSEARCH, quality-filtered, denoised, dereplicated and assigned to amplicon sequence variants by Deblur.

### Quantitative real-time PCR and reverse transcription PCR (RT-qPCR)

Gene abundance and expression (bacterial and archaeal 16S rRNA genes, fungal ITS, bacterial *ureC*, bacterial and archaeal *amoA*, *nxrA* from *Nitrobacter* and *Nitrospira* and *amoA* from comammox clade B) was estimated using quantitative real-time PCR (qPCR) and reverse transcriptase qPCR (RT-qPCR), respectively. Primer details are given in Table 1.

**Table 1:** Primers used for qPCR to assess gene abundance and activity

Gene	Primer	Sequence	Reference
16S rRNA Bacteria	341F	CCT AYG GGR BGC ASC AG	Glaring et al. (2015)
	806R	GGA CTA CNN GGG TAT CTA AT	
16S rRNA Archaea	arch349F	GYG CAS CAG KCG MGA AW	Takai and Horikoshi (2000)
	arch806R	GGA CTA CVS GGG TAT CTA AT	
16S rRNA Archaea	Parch519F	CAG CMG CCG CGG TAA	Ovreås et al. (1997)
	Arch1060R	GGC CAT GCA CCW CCT CTC	Reysenbach and Pace (1995)
ITS Fungi	ITS1f	TCC GTA GGT GAA CCT GCG G	Gardes and Bruns (1993)
	5.8 s	CGC TGC GTT CTT CAT CG	Vilgalys and Hester (1990)
*amoA* Bacteria	amoA-1F	GGG GTT TCT ACT GGT GGT	Rotthauwe et al. (1997)
	amoA-2R	CCC CTC KGS AAA GCC TTC TTC	
*amoA* Archaea	arch-amoAF	STA ATG GTC TGG CTT AGA CG	Francis et al. (2005)
	arch-amoAR	GCG GCC ATC CAT CTG TAT GT	
*ureC* Bacteria	ureC_Collier_F	AAG STS CAC GAG GAC TGG GGA	Collier et al. (1999)
	ureC_Collier_R	AGG TGG TGG CAS ACC ATS AGC AT	
*nxr*-*Nitrospira-*	nxr-spira-for5	CAR TCS AAC TTC CGG TAY GG	Fu et al. (2018)
	nxr-spira-rev6	AGC CAC TTG ATC ATG AAY TC	
*nxr*-*Nitrobacte*r	nxr-bacter-for1	GAC SCG YAC CCC SGA CGT GCA CYT CAT	
	nxr-bacter-rev3	ATG ACG TGR TTG RCC GCC ATC CA	
*amoA-comammox*	comaB-244F	TAY TTC TGG ACR TTY TA	Pjevac et al. (2017)
	comaB-659R	ARA TCC ARA CDG TGT G	

Amplifications were performed in 10 μl volumes containing 5 μl of QuantiFast SYBR Green PCR Master Mix for DNA and QuantiFast SYBR Green RT-PCR Master Mix for RNA (Qiagen, Manchester, UK), 0.1 μl of each primer (1 μM), 0.1 μl of QuantiFast RT Mix for RT-qPCR, 2 μl of template DNA at 5 ng μl^−1^ or 2–4 μl of RNA at 10 ng μl^−1^ and nuclease-free water (Severn Biotech, Kidderminster, UK) up to 10 μl, using a CFX384 Touch™ Real-Time PCR Detection System (Bio-Rad, Hemel Hempstead, UK). The amount of soil-extracted DNA or RNA added to each PCR reaction, at least 10 ng DNA or 20 ng RNA, is well above the 5 ng minimum recommended to avoid spurious results (Vestergaard et al. 2017).

The standards for each molecular target were obtained using a 10-fold serial dilution of PCR products amplified from an environmental reference DNA and purified by gel extraction using the Wizard® SV Gel and PCR Clean Up System (Promega, Southampton, UK) following the manufacturer’s instruction then quantified by fluorometer Qubit® 2.0 dsDNA BR Assay Kit (Thermo Fisher Scientific). Standard curve template DNA and the negative/positive controls were amplified in triplicate. Amplification conditions for all qPCR assays consisted of an initial denaturation at 95 °C for 5 min followed by 40 (two step) cycles, 95 °C for 10 s and 60 °C for 30 s. The RT-qPCR program had an initial reverse-transcription step at 50 °C for 10 min.

The conditions for comammox *amoA* clade B communities was adapted from Pjevac et al. (2017) to fit the constraints of the qPCR kit used but still matched the original conditions: initial denaturation at 95 °C for 5 min followed by 45 (three step) cycles, 95 °C for 30 s, 52 °C for 45 s and 72 °C for 1 min. Each amplification was followed by melt curve analysis (60 to 95 °C, with incremental readings every 0.5 °C) to assess the specificity of each assay. Results are expressed as gene copies per gram dw soil.

### Statistical analysis

GenStat 17th Edition (VSN International Ltd., Hemel Hempstead, UK) was used to perform one-way and General ANOVA to compare values obtained from soil analyses, grain and straw yield and N offtake and from qPCR estimations of gene and transcript copy numbers. To check that each set of measured values met the assumptions of ANOVA and were normally distributed, residuals were plotted. If they did not show normal distribution, data was log-transformed and again checked for normal distribution of residuals. Where ANOVA results were significantly different (*P <* 0.05), means were further tested using Tukey’s post hoc method in the GenStat multiple comparison menu with 95% confidence; significantly different means are considered to have α = 0.05 and are referred to as “significant” throughout the text. Where appropriate, the standard error of difference of means (s.e.d.) is indicated. Results with no significant differences are referred to as NSD.

The statistics package PAST v. 3.16 (Hammer et al. 2001) was used to perform NMDS with OTU data and Spearman’s rank correlation for soil properties and gene and transcript abundances at all sampling times.

## Results

### Soil pH, soil temperature, soil moisture and rainfall

**Fig. 1:**
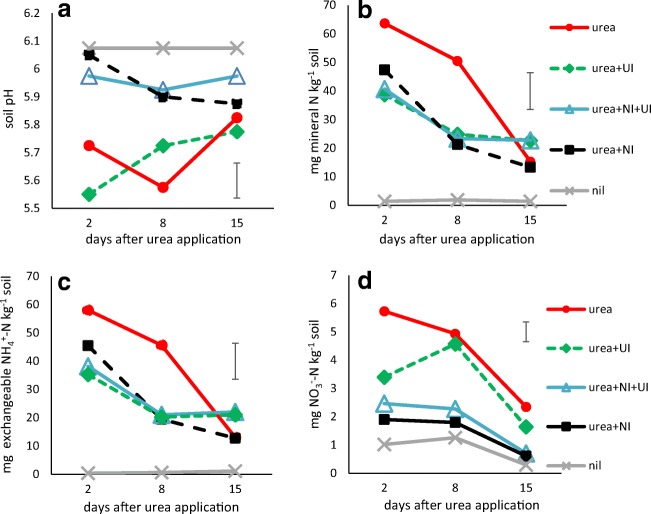
Soil edaphic factors measured at each sampling point (*n* = 4). All points were subjected to Tukey’s pot hoc test on ANOVA, significant results reported where *α* = 0.05. **a** soil pH, significantly lower in plots with urea or urea + UI. **b** Mineral N (exchangeable NH_4_^+^ + NO_3_^−^ + NO_2_^−^). **c** Exchangeable NH_4_^+^—in both, nil plot measurements significantly lower at each sampling time point than for any treatments. **d** NO_3_^−^, nil plots significantly lower and the urea and urea + UI plots significantly higher than plots with urea + NI or urea + NI + UI. ANOVA results are reported in Table 2

During the 16-day monitoring period, soil temperature at 10 cm was relatively stable, ranging from 7.5 to 10 °C (mean 9 °C). Rainfall of less than 2 mm was recorded on 5 days, making loss of urea or nitrate by leaching unlikely (supplementary Fig. 1). Prior to applying treatments, the field soil (previously reported to be 6.2) was measured at pH 6.1 in all designated plots and the nil plot soil remained at pH 6.1 throughout the monitoring period; the urea + NI and urea + NI + UI treatments were not significantly different but the plots with urea or urea + UI showed significantly lower pH at 2 and 8 days after application (Fig. 1a). ANOVA indicated that treatment, but not time since urea application, had a significant effect on pH (Table 2).

**Table 2:** ANOVA for soil edaphic factors at 2, 8 and 15 days after urea application from all samples (see Fig. 1). Mineral N = NO_3_^−^ + NO_2_^−^ + exchangeable NH_4_^+^

Source of variation	d.f.	pH	mineral N	NO_3_^−^	Exchangeable NH_4_^+^
Time	F_2, 45_	NS	8.17, *P* < 0.001	45.42, *P* < 0.001	3.61, *P* = 0.035
Treatment	F_4, 45_	10.79, *P* < 0.001	57.09, *P* < 0.001	31.9, *P* < 0.001	73.04, *P* < 0.001
Time × Treatment	F_8, 45_	NS	NS	NS	2.35, *P =* 0.033

*NS* not statistically significant

### Soil mineral N and crop yields

Total soil mineral N levels (exchangeable NH_4_^+^ + NO_3_^−^ + NO_2_^−^) in the nil plots were significantly lower than those where urea was applied, with or without inhibitors but there was NSD between these plots, with similar results for exchangeable NH_4_^+^ (Fig. 1b, c). The majority of mineral N in soil at 2 days was exchangeable NH_4_^+^, indicating rapid hydrolysis of urea that was not significantly affected by the presence of UI. However, on average, the NH_4_^+^ levels where urea was applied had declined 61% at 15 days after application, indicating active nitrification. Levels of NO_3_^−^ increased slightly 8 days after urea application but had decreased 40% at 15 days, with significantly less soil NO_3_^−^ observed where NI was applied with urea (Fig. 1d). ANOVA comparison of all samples for total mineral N, exchangeable NH_4_^+^ and NO_3_^−^, showed that both sampling time and treatment effects were significant, and interaction between these factors was significant for exchangeable NH_4_^+^ (Table 2). The total mineral N, NO_3_^−^ and NH_4_^+^ levels were strongly correlated (*r*_S_ = 0.62 and 0.99 respectively, *P* < 0.001—supplementary Table 3). The levels of NO_2_^−^ were too low and variable to infer statistical significance and are not reported.

**Fig. 2:**
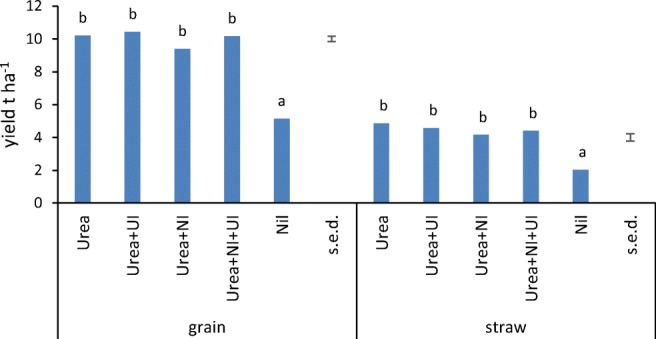
Wheat grain and straw yields at 85% dry matter expressed as t ha^−1^ (*n* = 4). Different letters above bars denote significantly different means (*α* = 0.05) according to Tukey’s post hoc test on ANOVA for each set of yields. ANOVA results: grain yield *F*_4,12_ = 95.0, *P* < .001; straw yield *F*_4,12_ = 29.7, *P* < .001

All plots with fertilizer addition yielded significantly higher than the nil plots for both grain and straw (Fig. 2a, b). There was a small but significant yield decrease in both grain and straw for urea + NI compared with urea, but not for the urea + UI or urea + NI + UI treatments. Results for grain and straw N offtake followed the same pattern (supplementary Fig. 2a, b). Significantly, more fertilizer N was recovered in straw and grain from ammonium nitrate fertilizer applied at the same N rate as the urea fertilizer treatments and compared with these, net N recovery from urea + NI was significantly less (supplementary Fig. 2c).

### Abundance and activity of soil microorganisms at kingdom level

A survey of the total bacterial community diversity in the different plots based on 16S rRNA amplicon sequencing showed no clear differences according to either sampling time or treatment (supplementary Fig. 3). The number of bacterial 16S rRNA genes was > 10-fold higher than the fungal ITS around 100× more than the archaeal 16S rRNA. Since there are thought to be on average 4–5 copies of the 16S rRNA gene in soil bacteria, 1–2 copies in soil archaea and an unknown number of ITS repeats, it is difficult to infer the actual cell numbers in each group. In contrast, the functional genes (*amoA*, *nxr*, *ureC*) generally have a single copy per genome. However, within-group comparisons show that the abundance of all genes had fallen 15 days after urea application, most of them significant (supplementary Fig. 4; Table 3). The treatment, however, did not have a significant effect on abundance except for bacterial *amoA* (Table 3). The number of transcripts also showed a significant response to time but not to treatment and indicated that bacteria were 5-fold more active than archaea and 50-fold more active than fungi (supplementary Fig. 5) and that activity increased over the 15-day period. ANOVA confirmed that sampling time, but not treatment, had a significant effect on all these measurements, *P* ≤ 0.05 (Table 3). Although the variability in efficiency of different PCR primers means that abundance estimates are not absolute but relative, there was no indication of PCR inhibitors in the DNA and RNA preparations as their amplification profiles matched those of the standard curves (supplementary Tables 1 and 2).

**Table 3:** ANOVA for gene and transcript copies in all treatments and times (see Supplementary Figs. 4 and 5). Time had a significant effect on most genes, whilst fertilizer treatment did not, affecting only the AOB which were significantly more abundant and active where urea was applied without NI

Gene copies g^−1^ soil	d.f.	16S Bacteria	16S Archaea	ITS	AOA *amoA*	AOB *amoA*	*ureC*	*nxr*-*bacter*	*nxr*-*spira*	Comammox
Time	F _2, 51_	5.13, *P* = 0.010	NS	4.96, *P* = 0.011	NS	19.96, *P* < 0.001	4.10, *P* = 0.023	18.49, *P* < 0.001	NS	NS
Treatment	F_4, 51_	NS	NS	NS	NS	8.71, *P* < 0.001	NS	NS	NS	NS
Time × treat	F_8, 51_	NS	NS	NS	NS	NS	NS	NS	NS	NS
Transcripts g^−1^ soil	d.f.	16S bacteria	16S archaea	ITS	AOA *amoA*	AOB *amoA*				
Time	F _2, 51_	21.58, *P* < 0.001	27.83 *P* < 0.001	13.29, *P* < 0.001	28.50, *P* < 0.001	23.68, *P* = 0.006				
Treatment	F_4, 51_	NS	NS	NS	NS	33.97, *P* = 0.003				
Time × treat	F_8, 51_	NS	NS	NS	NS	NS				

*NS* not statistically significant

The results indicate that the three kingdoms (Bacteria, Archaea, Fungi) increased transcriptional activity over the monitoring period whilst declining in abundance. The abundance of all three groups was strongly correlated, indicating similar responses to changes in soil conditions (supplementary Table 4).

### Abundance and activity of microorganisms involved in N-cycling

The genes involved with N-cycling, apart from bacterial *amoA*, showed a similar pattern to those at kingdom-level, with no significant treatment response but a significant decline over the 15-day monitoring period and moderate to strong correlation in all samples (supplementary Fig. 4, supplementary Table 3). The order of abundance of N-cycling genes was bacterial *ureC* > bacterial *amoA* > *Nitrospira nxr* > archaeal *amoA* > *Nitrobacter nxr* (supplementary Fig. 4). The proportion of AOB appeared to be relatively high compared with previous reports, at > 1% of total Bacteria; the AOA were > 20% of total Archaea. The under-representation of *ureC* compared with previous reports may be due to suboptimal primers for soil communities, although assuming it is a single copy gene and there are 5 copies of 16S rRNA genes per bacterial genome, it is present in c. 10% of soil bacteria and there is a strong correlation in abundance of 16S rRNA genes and *ureC* (*r*_s_ = 0.69, *P* < 0.001, supplementary Table 3). The PCR product from the comammox *amoA* clade B primers gave the wrong melting temperature (*T*_m_) and a double peak in the melting curves. These products gave a smear of multiple bands when run on a gel indicating that there was not a single specific product, in contrast to the other PCR assays. Because it was unclear which genes the primers were amplifying, the comammox results were disregarded. RNA extraction from the soils gave insufficient yields to detect transcription of the genes, apart from *amoA*, where the archaeal version increased over time, whereas there was a drop in bacterial *amoA* expression (supplementary Fig. 5). ANOVA showed sampling time to be significant (*P* ≤ 0.05) for most genes but not for the Archaea, AOA *amoA* or *Nitrospira nxr*, and treatment effects were significant only for AOB *amoA* (Table 3). The abundance of archaeal 16S RNA was strongly correlated with that of AOA *amoA* (*r*_s_ = 0.91, *P* << 0.001), whereas bacterial 16S RNA showed only a weak negative correlation with AOB *amoA* RNA (*r*_s_ = − 0.29, *P* = 0.03, supplementary Table 3).

### Responses of bacterial AOB to urea and inhibitors

**Fig. 3:**
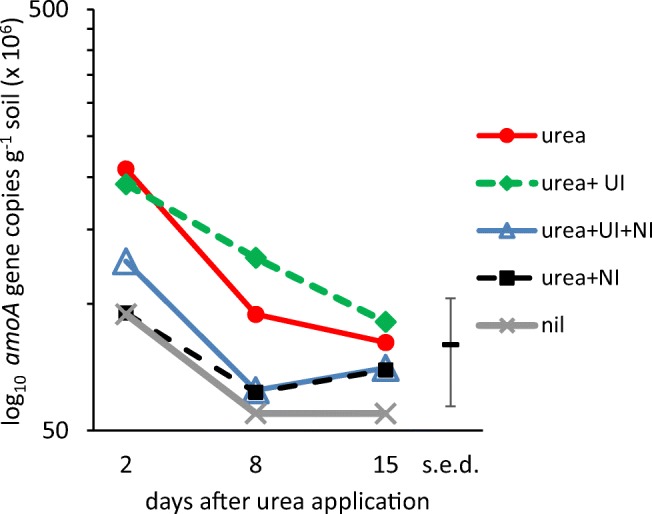
Abundance of bacterial *amoA* gene copies estimated using qPCR (*n* = 4) at 2, 8 and 15 days after application of urea fertilizer alone or in combination with nitrification inhibitor DCD (NI) and/or urease inhibitor NBPT (UI). Nil, no urea control. All points were subjected to Tukey’s pot hoc test on ANOVA, significant results reported where *α* = 0.05. The abundance of *amoA* in soils treated with urea alone or in combination with UI fell significantly between 2 and 8 days and was significantly greater than the other treatments at these days. ANOVA results are reported in Table 3

**Fig. 4:**
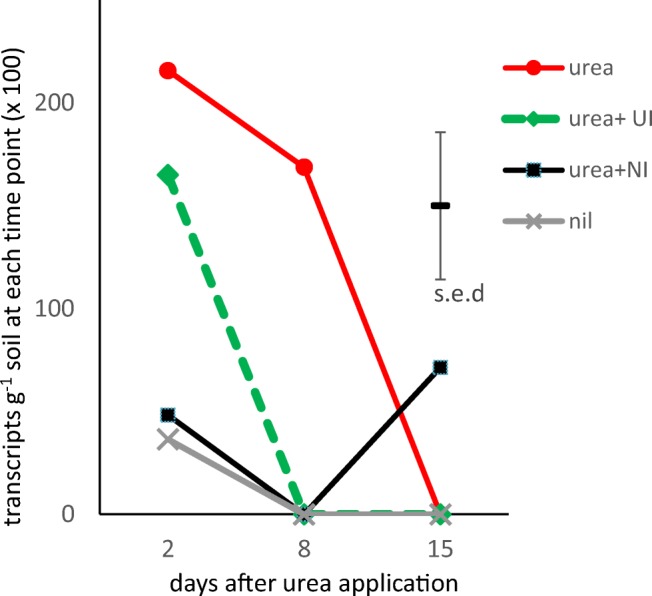
Abundance of bacterial *amoA* transcripts estimated using qPCR (*n* = 4) at 2, 8 and 15 days after application of urea fertilizer alone or in combination with nitrification inhibitor DCD (NI) and/or urease inhibitor NBPT (UI). Nil, no urea control. The soils treated with urea + NI + UI did not yield sufficient mRNA to be included in this figure and mRNA recovery was too low to infer statistical significance. ANOVA results are reported in Table 3

AOB abundance dropped over the sampling period in all treatments. For the urea and urea + UI treatment, AOB numbers remained significantly higher than those receiving NI at 2 and 8 days after urea application (Fig. 3). The transcript numbers were low and variable, and no statistical significance could be inferred but gene transcription was noticeably higher in the urea and urea + UI treatments (Fig. 4).

## Discussion

At the end of the experiment, grain and straw yields from the nil plots were less than half of those where N fertilizer was applied and the plots receiving ammonium nitrate yielded significantly more grain than plots receiving the same rate of N as urea (Fig. 2, supplementary Fig. 2), most likely because of a higher ammonia volatilization loss from the urea (Chambers and Dampney 2009). However, no yield enhancement was observed for any of the urea + inhibitor treatments, and the urea + NI treatment was associated with a small but significant reduction in N offtake and net N recovery in straw and grain when compared with urea (Fig. 2, supplementary Fig. 2). The reasons for this are unclear but may be related to the lower soil nitrate content in the period following application (Fig. 1). It is possible that delayed ammonia oxidation meant that when the bulk of the urea fertilizer was converted to hydroxylamine and nitrite, soil conditions were more conducive to biotic reduction to N_2_O resulting in net N loss from the system, compared with the other treatments but we could not confirm this as we were unable to measure gaseous losses during these experiments. The soils ranged between pH 5.5 and 6.1, insufficient acid for spontaneous decomposition of hydroxylamine to play a major role (Heil et al. 2016). It is possible that some degradation of the UI occurred: NBPT is reported to have a half-life of 1.6 days in soils at pH 5, 9.8 days at pH 5.5 and 42 days at pH 6 (Engel et al. 2015)*.* However, most ureolysis is likely to have occurred within 2 days (Nannipieri et al. 1990), before significant decomposition of the UI. Surprisingly, yields and net N uptake were not significantly diminished when NI was combined with UI but remained similar to urea alone or urea with just UI. Whilst the meta-analysis of Abalos et al. (2014) showed predominantly positive impacts of NI use on crop yields, yield suppression has also been observed (e.g. Bell et al. 2015), which may be related to the timing of availability of different forms of soil N in relation to plant uptake.

There are conflicting reports in the literature on UI influence on crop yield (Sigurdarson et al. 2018). For example, the UI phenylphosphoryldiamidate (PPDA) was found to have no effect on wheat yields in Syria (Monem et al. 2010), but in tropical soils, NBPT was reported to reduce urea hydrolysis by 35% and, in conjunction with a NI, increases maize yields significantly (Martins et al. 2017). In a large UK study, Chambers and Dampney (2009) reported a mean ammonia emission reduction of 70% (range 25–100%) from the use of NBPT with urea, and on average the use of NBPT increased crop N recovery compared with urea alone. However, differences at an individual site were not always significant and this may indicate lack of effectiveness of the UI due to rapid breakdown in soil under certain conditions.

The Woburn soil is slightly acidic and well-drained, and the experiment took place in a period of low rainfall, although it rained on the day that treatments were applied. Sampling of the field was constrained by practical considerations: the farm is run on commercial lines which dictate timing of treatments and access for sampling. In retrospect, it would have been informative to sample on the day following application. The soil pH was slightly lower 2 days after application, where urea and urea + UI were applied, compared with nil plots and those with NI. This indicates a very rapid hydrolysis of urea to NH_4_^+^ and subsequent nitrification. The finding is supported by reports of an experiment where ^15^N-labelled urea was applied to a grass and legume sward under Mediterranean conditions: ureolysis occurred within 2 days and ^15^N-NH_4_^+^ peaked at 2 days (Nannipieri et al. 1990). Urea can increase soil pH as hydrolysis to NH_4_^+^ releases one OH^−^ but subsequent nitrification to NO_3_^−^ releases two H^+^, resulting in net soil acidification. The presence of UI did not retard acidification, but pH in soil where NI was added was similar to the nil control, indicating less acidification due to delayed ammonia oxidation. The mineral N and exchangeable NH_4_^+^ concentrations in soil were similar during the experiment confirming that most urea was already hydrolysed by the first sampling and the drop in pH was a residual effect of urea hydrolysis followed by nitrification. The mineral N concentrations also were higher in plots with urea alone, compared with the various inhibitor combinations, but this was not statistically significant and only the nil plot had significantly less N. However, the presence of NI resulted in significantly lower NO_3_^−^, indicating an effect over 2 weeks. The rate of nitrification in soil is reported to be less rapid than urea hydrolysis: a meta-analysis reported nitrification rates of 1.4–2 μg NH_4_^+^-N g soil^−1^ day^−1^ (Booth et al. 2005). This compares with rates of 5 μg–6 mg urea-N kg soil^−1^ day^−1^ for urea measured in a range of moist soil (Reynolds et al. 1985). With lower NO_3_^−^ concentrations in soil, less N2O will be emitted due to denitrification. We did not measure gaseous losses in the field but experiments with the NI 3,4-dimethylpyrazole phosphate (DMPP) indicated that it resulted in lower N_2_O emissions (Zhang et al. 2018). It would be interesting to determine whether the inhibitors have any beneficial environmental effects in the Woburn soil by decreasing NH_3_ or N_2_O emissions. Otherwise, the lack of any significant yield increases with NI and UI (singly or in combination) negates any economic case for their use in this situation.

The abundance of soil microorganisms at kingdom level (bacteria, archaea, fungi) fell during the monitoring period, and gene expression increased, presumably a response to an earlier stimulation due to temperatures, rainfall and plant growth. The bacterial urease, archaeal AMO and NXR genes showed the same pattern of a drop-in abundance and increase in activity, indicating a common trend in the soil community responding to environmental factors but not to the different treatments which were NSD.

In arable soils receiving N fertilizer, AOB have been reported to be more active than AOA (Hink et al. 2017). In our experiment, the AOB increased in both abundance and activity in response to urea or urea + UI applications despite insignificant differences in the levels of exchangeable NH_4_^+^; the presence of NI reduced this effect. This indicates that inhibition of AMO affected AOB growth even when differences in the substrate NH_4_^+^ were not discernible. A drop in the abundance of AOB *amoA* has been reported in Australian sugarcane soils treated with the NI DMPP (Zhang et al. 2018). Since AOB numbers were already significantly higher 2 days after urea application, it is likely that the NH_4_^+^ levels in soil resulting from urea hydrolysis had increased very rapidly after application and were falling due to AOB activity at this first sampling date. The larger AOB community in the urea and urea + UI plots appeared to result in more NO_3_^−^ as well as lower soil pH as mentioned above. Although the NOB must have been actively oxidizing NO_2_^−^ to NO_3_^−^, no effects on their abundance were detected. The AOB *amoA* primers are not expected to amplify comammox *amoA* (Pjevac et al. 2017) and it is unlikely that the comammox bacteria were major contributors to nitrification in the soil as they are only a sub-population of the *Nitrospira*, in turn 70% less abundant than the AOB. To monitor comammox in these soils, it will be necessary to develop new primers for PCR with improved *amoA* specificity.

## Conclusions

For the winter wheat crop on sandy loam at Woburn in 2017, the addition of the UI NBPT and the NI DCD had only transient effects on soil N dynamics and did not result in increased crop yields. It is likely that urea hydrolysis by extracellular and intracellular enzymes was very rapid, followed by nitrification due to AOB and NOB activity. There were no discernible effects on soil microbial community dynamics, whether bacteria, archaea or fungi, nor on urease gene frequency, ammonia-oxidizing archaea or nitrite-oxidizing bacteria. However, ammonia-oxidizing bacteria numbers increased in response to urea and urea + UI, less so when NI was present, indicating that the UI had only a short-lived effect within the first two days on the supply of the NH_4_^+^ substrate for AOB. The lack of response from AOA and NOB implies that services provided by these groups are largely unaffected by soil treatments and furthermore that growth of AOA in soil is not inhibited by DCD. In conclusion, with a caveat that our findings may not apply to other soils, crops and climates, the UI NBPT and the NI DCD had only minor effects on soil pH, N dynamics and AOB with no discernible influence on other soil microorganisms and no positive effects on crop yields.

## Electronic supplementary material

ESM 1 (DOCX 90 kb)

## References

[CR1] Abalos D, Jeffery S, Sanz-Cobena A, Guardia G, Vallejo A (2014). Meta-analysis of the effect of urease and nitrification inhibitors on crop productivity and nitrogen use efficiency. Agric Ecosyst Environ.

[CR2] Akiyama H, Yan XY, Yagi K (2010). Evaluation of effectiveness of enhanced-efficiency fertilizers as mitigation options for N2O and NO emissions from agricultural soils: meta-analysis. Glob Chang Biol.

[CR3] Amberger A (2008). Research on dicyandiamide as a nitrification inhibitor and future outlook. Commun Soil Sci Plant Anal.

[CR4] Bell MJ, Hinton N, Cloy JM, Topp CFE, Rees RM, Cardenas L, Scott T, Webster C, Ashton RW, Whitmore AP, Williams JR, Balshaw H, Paine F, Goulding KWT, Chadwick DR (2015). Nitrous oxide emissions from fertilised UK arable soils: fluxes, emission factors and mitigation. Agric Ecosyst Environ.

[CR5] Booth MS, Stark JM, Rastetter E (2005). Controls on nitrogen cycling in terrestrial ecosystems: a synthetic analysis of literature data. Ecol Monogr.

[CR6] Chambers BJ, Dampney PMR (2009). Nitrogen efficiency and ammonia emissions from urea-based and ammonium nitrate fertilisers. Proc Intl Fert Soc.

[CR7] Collier JL, Brahamsha B, Palenik B (1999). The marine cyanobacterium Synechococcus sp. WH7805 requires urease (urea amidohydrolase, EC 3.5.1.5) to utilize urea as a nitrogen source: molecular-genetic and biochemical analysis of the enzyme. Microbiology-SGM.

[CR8] Daims H, Lucker S, Wagner M (2016). A new perspective on microbes formerly known as nitrite-oxidizing bacteria. Trends Microbiol.

[CR9] Delmont TO, Robe P, Cecillon S, Clark IM, Constancias F, Simonet P, Hirsch PR, Vogel TM (2011). Accessing the soil metagenome for studies of microbial diversity. Appl Environ Microbiol.

[CR10] Engel RE, Towey BD, Gravens E (2015). Degradation of the urease inhibitor NBPT as affected by soil pH. Soil Sci Soc Am J.

[CR11] FAO (1990) Guidelines for Soil Profile Description, 3rd edition. Food and Agriculture Organization of the United Nations, Rome

[CR12] Francis CA, Roberts KJ, Beman JM, Santoro AE, Oakley BB (2005). Ubiquity and diversity of ammonia-oxidizing archaea in water columns and sediments of the ocean. Proc Natl Acad Sci U S A.

[CR13] Fu QL, Clark IM, Zhu J, Hu HQ, Hirsch PR (2018). The short-term effects of nitrification inhibitors on the abundance and expression of ammonia and nitrite oxidizers in a long-term field experiment comparing land management. Biol Fertil Soils.

[CR14] Gardes M, Bruns TD (1993). ITS primers with enhanced specificity for basidiomycetes--application to the identification of mycorrhizae and rusts. Mol Ecol.

[CR15] Gilsanz C, Baez D, Misselbrook TH, Dhanoa MS, Cardenas LM (2016). Development of emission factors and efficiency of two nitrification inhibitors, DCD and DMPP. Agric Ecosyst Environ.

[CR16] Glaring Mikkel A., Vester Jan K., Lylloff Jeanette E., Abu Al-Soud Waleed, Sørensen Søren J., Stougaard Peter (2015). Microbial Diversity in a Permanently Cold and Alkaline Environment in Greenland. PLOS ONE.

[CR17] Hammer Ø, Harper DAT, Ryan PD (2001). Past: paleontological statistics software package for education and data analysis. Palaeontol Electron.

[CR18] Heil J, Vereecken H, Bruggemann N (2016). A review of chemical reactions of nitrification intermediates and their role in nitrogen cycling and nitrogen trace gas formation in soil. Eur J Soil Sci.

[CR19] Hink L, Nicol GW, Prosser JI (2017). Archaea produce lower yields of N_2_O than bacteria during aerobic ammonia oxidation in soil. Environ Microbiol.

[CR20] Hirsch PR, Jhurreea D, Williams JK, Murray PJ, Scott T, Misselbrook TH, Goulding KWT, Clark IM (2017). Soil resilience and recovery: rapid community responses to management changes. Plant Soil.

[CR21] Johnston AE, Poulton PR, Coleman K, Macdonald AJ, White RP (2017). Changes in soil organic matter over 70 years in continuous arable and ley-arable rotations on a sandy loam soil in England. Eur J Soil Sci.

[CR22] Klose S, Tabatabai MA (1999). Urease activity of microbial biomass in soils. Soil Biol Biochem.

[CR23] Kowalchuk GA, Stephen JR (2001). Ammonia-oxidizing bacteria: a model for molecular microbial ecology. Annu Rev Microbiol.

[CR24] Lloyd AB, Sheaffe MJ (1973). Urease activity in soils. Plant Soil.

[CR25] Martins MR, Sant’Anna SAC, Zaman M, Santos RC, Monteiro RC, Alves BJR, Jantalia CP, Boddey RM, Urquiaga S (2017). Strategies for the use of urease and nitrification inhibitors with urea: impact on N_2_O and NH_3_ emissions, fertilizer-N-15 recovery and maize yield in a tropical soil. Agric Ecosyst Environ.

[CR26] Monem MA, Lindsay WL, Sommer R, Ryan J (2010). Loss of nitrogen from urea applied to rainfed wheat in varying rainfall zones in northern Syria. Nutr Cycl Agroecosyst.

[CR27] Nannipieri P, Ciardi C, Palazzi T, Badalucco L (1990). Short-term nitrogen reactions following the addition of urea to a grass legume association. Soil Biol Biochem.

[CR28] Nannipieri P, Trasar-Cepeda C, Dick RP (2018). Soil enzyme activity: a brief history and biochemistry as a basis for appropriate interpretations and meta-analysis. Biol Fertil Soils.

[CR29] Ovreås L, Forney L, Daae FL, Torsvik V (1997). Distribution of bacterioplankton in meromictic Lake Saelenvannet, as determined by denaturing gradient gel electrophoresis of PCR-amplified gene fragments coding for 16S rRNA. Appl Environ Microbiol.

[CR30] Parada AE, Needham DM, Fuhrman JA (2016). Every base matters: assessing small subunit rRNA primers for marine microbiomes with mock communities, time series and global field samples. Env Microbiol.

[CR31] Pjevac P, Schauberger C, Poghosyan L, Herbold CW, van Kessel M, Daebeler A, Steinberger M, Jetten MSM, Lucker S, Wagner M, Daims H (2017). *AmoA-*targeted polymerase chain reaction primers for the specific detection and quantification of comammox *Nitrospira* in the environment. Front Microbiol.

[CR32] Prosser JI, Nicol GW (2012). Archaeal and bacterial ammonia-oxidisers in soil: the quest for niche specialisation and differentiation. Trends Microbiol.

[CR33] Qin SP, Hu CS, Wang YY, Li XX, He XH (2010). Tillage effects on intracellular and extracellular soil urease activities determined by an improved chloroform fumigation. Method Soil Sci.

[CR34] Quince C, Lanzen A, Davenport RJ, Turnbaugh PJ (2011). Removing noise from pyrosequenced amplicons. BMC Bioinformatics.

[CR35] Reynolds CM, Wolf DC, Armbruster JA (1985). Factors related to urea hydrolysis in soils. Soil Sci Soc Am J.

[CR36] Reysenbach AL, Pace NR, Rob FT (1995). Reliable amplification of hyperthermophilic archaeal 16S rRNA genes by the polymerase chain reaction. Archaea: a laboratory manual.

[CR37] Rose TJ, Wood RH, Rose MT, Van Zwieten L (2018). A re-evaluation of the agronomic effectiveness of the nitrification inhibitors DCD and DMPP and the urease inhibitor NBPT. (vol 252, pg 69, 2017). Agric Ecosyst Environ.

[CR38] Rotthauwe JH, Witzel KP, Liesack W (1997). The ammonia monooxygenase structural gene *amoA* as a functional marker: molecular fine-scale analysis of natural ammonia-oxidizing populations. Appl Environ Microbiol.

[CR39] Sigurdarson JJ, Svane S, Karring H (2018). The molecular processes of urea hydrolysis in relation to ammonia emissions from agriculture. Rev Environ Sci Biotechnol.

[CR40] Silva AGB, Sequeira CH, Sermarini RA, Otto R (2017). Urease inhibitor NBPT on ammonia volatilization and crop productivity: a meta-analysis. Agron J.

[CR41] Takai K, Horikoshi K (2000). Rapid detection and quantification of members of the archaeal community by quantitative PCR using fluorogenic probes. Appl Environ Microbiol.

[CR42] Vestergaard G, Schulz S, Scholer A, Schloter M (2017). Making big data smart-how to use metagenomics to understand soil quality. Biol Fertil Soils.

[CR43] Vilgalys R, Hester M (1990). Rapid genetic identification and mapping of enzymatically amplified ribosomal DNA from several *Cryptococcus* species. J Bacteriol.

[CR44] Wrage-Monnig N, Horn MA, Well R, Muller C, Velthof G, Oenema O (2018). The role of nitrifier denitrification in the production of nitrous oxide revisited. Soil Biol Biochem.

[CR45] Zaman M, Nguyen ML, Blennerhassett JD, Quin BF (2008). Reducing NH_3_, N_2_O and NO_3_^-^-N losses from a pasture soil with urease or nitrification inhibitors and elemental S-amended nitrogenous fertilizers. Biol Fertil Soils.

[CR46] Zhang MY, Wang WJ, Tang L, Heenan M, Xu ZH (2018). Effects of nitrification inhibitor and herbicides on nitrification, nitrite and nitrate consumptions and nitrous oxide emission in an Australian sugarcane soil. Biol Fertil Soils.

