# Supplementary material for: Effects of urease and nitrification inhibitors on soil N, nitrifier abundance and activity in a sandy loam soil
Source: Biol Fertil Soils. 2019 Nov 25;56(2):185–94. doi: 10.1007/s00374-019-01411-5 (PMC6981326; doi:10.1007/s00374-019-01411-5)
Supplement: Supplementary file 1 — (DOCX 90 kb) [file 374_2019_1411_MOESM1_ESM.docx]

**Supplementary Fig. 1.** Daily measurements at Woburn of soil temperature (10 cm depth) and rainfall after urea application.


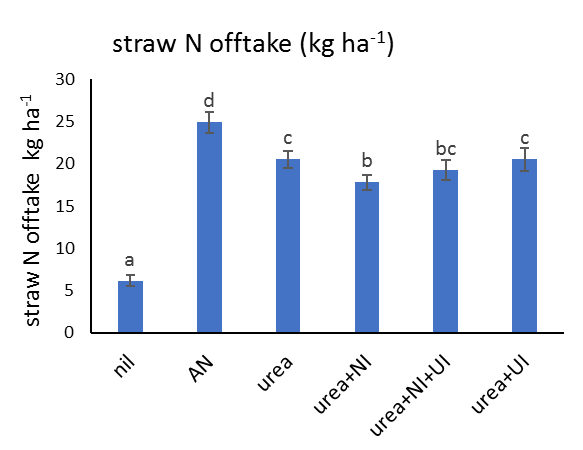

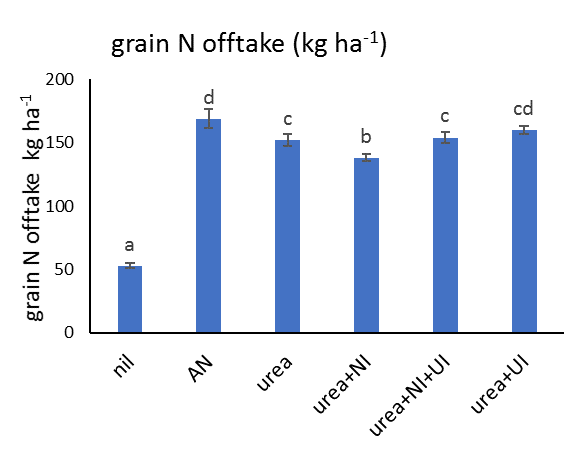
 **Supplementary Fig. 2**. Wheat grain and straw N offtake and net N recovery in all treatment plots (n = 6). Letters above columns denote significantly different means according to Tukey’s post-hoc test on ANOVA, α = 0.05. AN – ammonium nitrate fertilizer applied at same N rate as urea.


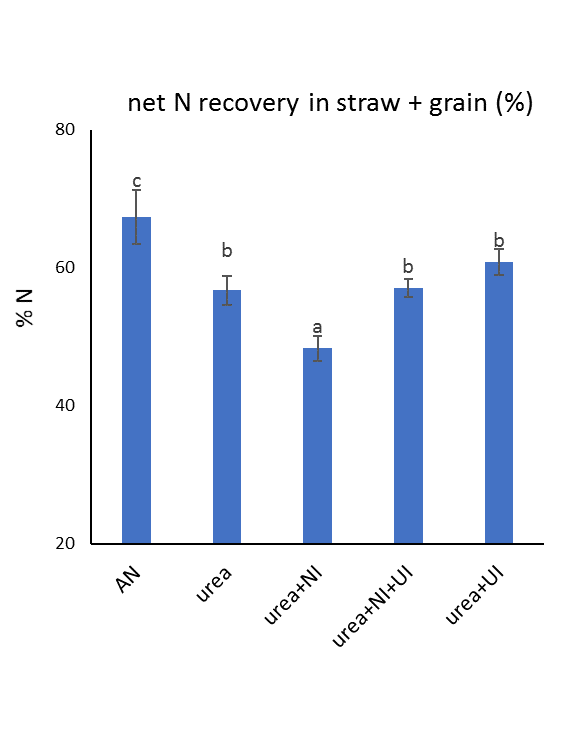
**Supplementary Fig. 3.** NMDS plot, Bray-Curtis matrix, for 16S rRNA amplicon diversity (2000 most abundant OTU) in all treatments 2 d after urea application.

**Supplementary Fig. 4.** Mean abundance for all treatments of genes at each time point. All genes were significantly less abundant at 15 days compared to two days after urea addition (Tukey’s post-hoc *α* = 0.05) except *nxr Nitrospira* and *amoA* Archaea (NSD).

**Supplementary Fig. 5.** Mean abundance for all treatments of transcripts at each time point. All transcripts were significantly more abundant at 15 days compared to two days after urea addition except *amo* Bacteria which was significantly less abundant (Tukey’s post-hoc *α* = 0.05).

**Supplementary Table 1. PCR efficiency of primer sets**

|  | **Efficiency** | **r²** | **slope** | **y-int** |
| --- | --- | --- | --- | --- |
| **16S Bacteria** | 84.8% | 0.995 | -3.749 | 36.359 |
| **ITS Fungi** | 87.30% | 0.998 | -3.669 | 35.258 |
| **16S Archaea** | 100.10% | 0.995 | -3.319 | 33.365 |
| ***ureC* Bacteria** | 95.40% | 0.997 | -3.437 | 31.779 |
| ***amoA* Bacteria** | 82.20% | 0.997 | -3.837 | 35.908 |
| ***nxr Nitrospira*** | 92.70% | 0.999 | -3.511 | 31.665 |
| ***amoA* Archaea** | 86% | 0.997 | -3.709 | 33.318 |
| ***nxr Nitrobacter*** | 93.30% | 0.999 | -3.494 | 33.457 |

**Supplementary Table 2. PCR efficiency of primer sets in RNA RT-qPCR**

|  | **Efficiency** | **r²** | **slope** | **y-int** |
| --- | --- | --- | --- | --- |
| **16S Bacteria** | 78.8% | 0.999 | -3.962 | 36.922 |
| **16S Archaea** | 93.40% | 0.995 | -3.491 | 33.852 |
| **ITS Fungi** | 91.90% | 0.998 | -3.532 | 34.095 |
| ***amoA* Archaea** | 90% | 0.997 | -3.601 | 32.279 |
| ***amoA* Bacteria** | 90.50% | 0.999 | -3.572 | 34.172 |

| **NO_3_^-^** | -0.46 |  |  |  |  |  |  |  |  |  |  |  |  |  | |  | |  |  |
| --- | --- | --- | --- | --- | --- | --- | --- | --- | --- | --- | --- | --- | --- | --- | --- | --- | --- | --- | --- |
| **NH_4_^+^** | -0.46 | 0.55 |  |  |  |  |  |  |  |  |  |  |  |  |  | |  | | |
| **mineral N** | -0.44 | **0.62** | **0.99** |  |  |  |  |  |  |  |  |  |  |  |  | |  | | |
| **16S Bacteria** | -0.02 | 0.21 | 0.18 | 0.19 |  |  |  |  |  |  |  |  |  |  |  | |  | | |
| **16S Archaea** | 0.08 | 0.17 | 0.12 | 0.13 | **0.76** |  |  |  |  |  |  |  |  |  |  | |  | | |
| **ITS Fungi** | -0.25 | 0.32 | 0.36 | 0.36 | **0.61** | **0.66** |  |  |  |  |  |  |  |  |  | |  | | |
| ***ureC* Bacteria** | -0.06 | 0.18 | 0.18 | 0.19 | **0.69** | **0.85** | **0.76** |  |  |  |  |  |  |  |  | |  | | |
| ***amoA* Bacteria** | -0.38 | **0.64** | 0.46 | 0.51 | 0.49 | 0.43 | **0.62** | 0.46 |  |  |  |  |  |  |  | |  | | |
| ***amoA* Archaea** | 0.13 | -0.02 | -0.04 | -0.03 | 0.36 | **0.64** | 0.27 | 0.48 |  |  |  |  |  |  |  | |  | | |
| ***nxrA Nitrospira*** | -0.06 | 0.06 | 0.13 | 0.13 | **0.70** | **0.81** | **0.75** | **0.82** | 0.46 | 0.59 |  |  |  |  |  | |  | | |
| ***nxrA Nitrobacter*** | -0.10 | 0.21 | 0.24 | 0.25 | **0.66** | **0.72** | **0.70** | **0.74** | **0.62** | 0.34 | **0.81** |  |  |  |  | |  | | |
| **16S RNA Bacteria** | -0.02 | -0.35 | -0.14 | -0.19 | -0.25 | -0.24 | -0.30 | -0.30 | -0.36 | 0.07 | -0.24 | -0.41 |  |  |  | |  | | |
| **16S RNA Archaea** | -0.11 | -0.18 | -0.08 | -0.11 | -0.17 | -0.15 | -0.23 | -0.21 | -0.28 | 0.11 | -0.13 | -0.30 | **0.75** |  |  | |  | | |
| **ITS RNA Fungi** | -0.23 | -0.05 | 0.05 | 0.03 | -0.21 | -0.21 | -0.14 | -0.22 | -0.13 | -0.01 | -0.18 | -0.26 | **0.65** | **0.87** |  | |  | | |
| ***amoA* RNA AOB** | -0.41 | 0.37 | 0.25 | 0.27 | 0.28 | 0.25 | 0.30 | 0.29 | 0.39 | 0.04 | 0.15 | 0.33 | -0.29 | -0.23 | -0.15 | |  | | |
| ***amoA* RNA AOA** | -0.19 | -0.04 | 0.00 | -0.01 | -0.06 | -0.06 | -0.09 | -0.11 | -0.22 | 0.13 | -0.08 | -0.24 | **0.61** | **0.91** | **0.84** | | -0.09 | | |
|  | **pH** | **NO_3_^-^** | **NH_4_^+^** | **min N** | **16S Bact** | **16S Arch** | **ITS Fun** | ***ureC* Bact** | ***amoA* AOB** | ***amoA* AOA** | ***nxrA spira*** | ***nxrA bacter*** | **16S**  **RNA Bact** | **16S**  **RNA Arch** | **ITS**  **RNA Fungi** | | ***amoA* RNA AOB** | |  |

**Supplementary Table 3.**  Spearman’s rank correlation (*r*_s_) for all samples, plots and times. Statistically significant correlations (*P* <0.05) are highlighted yellow; strong positive or negative correlations (>.6) are in **bold.**
